# Implementation of a clinical decision support tool to improve the adequate prescription of low-molecular-weight heparins in non-surgical patients

**DOI:** 10.1371/journal.pone.0350017

**Published:** 2026-06-08

**Authors:** Froucke van Gosliga, Dagmar Pals, Annette van Ojik, Eric van Roon

**Affiliations:** 1 Center of Pharmacy, Frisius Medical Center, Leeuwarden, The Netherlands; 2 Faculty of Science and Engineering, University of Groningen, Groningen, The Netherlands; Tokyo Women’s Medical University: Tokyo Joshi Ika Daigaku, JAPAN

## Abstract

**Background:**

Non-surgical hospitalized patients have an increased risk of developing venous thromboembolism (VTE). This risk can be reduced by thromboprophylaxis with low-molecular-weight heparins (LMWHs), but adherence to thromboprophylaxis guidelines is generally low.

**Objectives:**

To study the effect of implementing a clinical decision support (CDS) tool in the electronic health record on the percentage of adequately prescribed thromboprophylaxis with an LMWH in non-surgical patients at high risk for VTE.

**Methods:**

Data on the Padua Prediction Score (PPS) and thromboprophylaxis in high-risk non-surgical patients (≥18 years) were collected at different time points before and after the implementation of the intervention consisting of a validated CDS tool. The percentage of adequately prescribed LMWH thromboprophylaxis was described pre- and post-intervention in a stepwise approach, in which both crude and adjusted pre- and post-intervention differences were assessed using an interrupted time series analysis, accounting for potential time trends and autocorrelation.

**Results:**

In 400 patients included, 200 pre- and 200 post-intervention, the percentage of adequately prescribed LMWH thromboprophylaxis increased from 78% to 91%, an increase of 13% (95% CI: 6%−20%). This effect diminished to 8% (95% CI: −4%−19%) after adjustment for the pre-intervention slope and autocorrelation.

**Conclusion:**

In our study, the already high percentage of patients with adequately prescribed LMWH-thromboprophylaxis could potentially be increased further with the implementation of a CDS tool. With increasing amounts of data available in electronic health records, CDS tools might be an efficient and sustainable intervention to improve healthcare quality.

## Introduction

Venous thromboembolism (VTE), including deep vein thrombosis (DVT) and pulmonary embolism (PE), is the third most common cardiovascular diagnosis and a leading cause of increased healthcare costs and preventable death in hospitalized patients [1–3]. About 60% of all cases of VTE are hospital-acquired, occurring during or after a recent hospital admission [4]. Without adequate thromboprophylaxis, approximately 3% of hospitalized non-surgical patients develop VTE, increasing to 11% among patients at elevated risk for developing VTE [5]. The appropriate use of primary thromboprophylaxis in the form of a low-molecular-weight heparin (LMWH) in hospitalized non-surgical patients at increased risk for VTE has proven to be safe, effective, and cost-effective in reducing DVT and PE [5–7].

Although there is some discussion about which risk assessment model works best for identifying (subtypes of) patients at increased risk of VTE [8–13], the Padua Prediction Score (PPS), shown in Table 1, is currently considered the best available model to stratify between non-surgical patients with a high and low risk of VTE. Therefore, the PPS is included in international and national thromboprophylaxis guidelines to determine risk of VTE [5,14,15]. According to these guidelines, thromboprophylaxis, preferably in the form of an LMWH, should be started if a patient is at high risk for VTE.

**Table 1:** Padua Prediction Score risk assessment tool.

Risk factor	Score
Active cancer^a^	3
Previous VTE (with the exclusion of superficial vein thrombosis)	3
Reduced mobility^b^	3
Already known thrombophilic condition^c^	3
Recent (≤1 month) trauma and/or surgery	2
Elderly age (≥70 years)	1
Heart and/or respiratory failure	1
Acute myocardial infarction or ischemic stroke	1
Acute infection and/or rheumatologic disorder	1
Obesity (BMI ≥ 30)	1
Ongoing hormonal treatment	1

VTE: Venous thromboembolism; BMI: Body Mass Index.

^a^ Patients with local or distant metastases and/or in whom chemotherapy or radiotherapy had been performed in the previous 6 months.

^b^ Bedrest with bathroom privileges (either due to patient’s limitations or on physicians order) for at least 3 days.

^c^ Carriage of defects of antithrombin, protein C or S, factor V Leiden, G20210A prothrombin mutation, antiphospholipid syndrome.

However, adherence to thromboprophylaxis guidelines is low, and studies show that thromboprophylaxis continues to be used inadequately [16–20]. According to a Dutch report published in 2017, 49% of the non-surgical hospitalized patients with a high risk of VTE received adequate thromboprophylaxis according to the Dutch guideline [21], which was confirmed by a more recent Dutch study performed in 2021 [6].

A recent review assessing the effects of interventions to increase the adequate use of thromboprophylaxis in hospitalized patients found that alerts and multifaceted interventions were associated with increased use of thromboprophylaxis, with computer alerts suggested as the most effective intervention [22]. Computerized clinical decision support (CDS) systems are associated with substantial improvements in the use of adequate prophylaxis and reductions in VTE events, particularly in non-surgical patients [23,24]. The multifaceted intervention from Jaspers et al, increased thromboprophylaxis guideline adherence from 49% to 82%, which they believe can mostly be attributed to the implementation of a highly specific and user-friendly CDS tool integrated into the electronic health record (EHR) [6].

We aimed to study the effect of a single intervention, the implementation of a CDS tool in the electronic health record, on the percentage of adequately prescribed thromboprophylaxis in non-surgical patients with a high VTE risk. We looked at both the crude effect and the adjusted pre-post intervention effect that was corrected for potential underlying pre-intervention time trends and autocorrelation using an interrupted time series analysis.

## Methods

### Study design

This study was conducted at the Medical Centre Leeuwarden, a large teaching hospital in the Netherlands. The study has a prospective time series design with data collected at different time points pre- and post-intervention.

### Patient selection

Non-surgical patients ≥18 years of age who were admitted to a medical ward with a hospital stay of ≥48 hours were eligible for inclusion in the study. Patients with a high risk of VTE, defined as a PPS of ≥4 (Table 1), and no anticoagulant in use were included. Patients who had recently (<1 week) undergone a percutaneous coronary intervention and palliative patients were excluded.

### Intervention

The intervention consisted of the implementation of a CDS tool involving a Best Practice Advisory (BPA) in Epic® (Epic Systems, Verona, USA). This tool provides an automated recommendation to the prescriber of the appropriate specialization when there is an indication for thromboprophylaxis according to the PPS (score ≥4). The CDS tool collects data from the patient’s EHR on patient characteristics (sex, age, BMI), from the patient’s problem list (e.g., active cancer, previous VTE, thrombophilic condition), the active medication list (including oncology treatment plans),and the nursing assessment (mobility). The nursing assessment is carried out several times a day, and information about mobility (e.g., bedbound, sitting in chair, walking) is documented. When there is no anticoagulant in use at 36 hours after start of admission in the active medication list the recommendation is shown to the prescriber (physician, physician assistant or certified nurse specialist) of the main specialty as a pop-up when opening the patient file. The CDS tool pop-up displays the PPS and associated risk factors and an order for nadroparin 2,850 IU, the standard prophylactic dose according to local protocol, can be initiated immediately. The bleeding risk is not taken into account in the tool and has to be assessed by the prescriber. An action has to be taken and a reason has to be provided if the advice is not adopted by the prescriber, choosing from: I am not the responsible physician, the calculated score is not correct, there is a contraindication, or other (with explanation). Before live implementation, we tested the CDS tool in a test environment and evaluated its performance. The CDS tool achieved a sensitivity of 92% and a specificity of 68%. Prescribers were informed about the implementation of the new CDS tool before going live.

### Data collection

Data were collected between July 2022 and July 2023 and the CDS tool became operational in March 2023. Data were collected at ten different time points, with each time point representing approximately one month. Data were collected in Microsoft Excel 2021 from the first working day of the month until 40 patients per month were included.

### Statistical analysis

Patient characteristics were described before and after the intervention with median (interquartile range [IQR]) for continuous variables and numbers (%) for categorical variables. Patient characteristics were compared using the Mann-Whitney U test for continuous variables and the Fisher’s exact test for categorical variables.

The primary endpoint, the percentage of adequately prescribed thromboprophylaxis, was first assessed for the combined time points before and after the intervention with a Fisher’s exact test and the pre-post difference with the 95% confidence interval (CI) was reported. Next the potential pre-implementation time trend was determined and corrected for by using an interrupted time series (ITS) analysis using an autoregressive integrated moving average (ARIMA) model, both unadjusted and adjusted for autocorrelation.

At the time the study was designed, no standard methodological guidance was available for power or sample size considerations within an ITS framework. Therefore, no formal sample size calculation was performed. Given the exploratory nature of the study, p-values are not provided and estimates are presented with appropriate indicators of uncertainty. Statistical analysis was performed using SPSS Statistics 28.0 (IBM, New York, USA).

### Ethical approval

Local feasibility of the study was assessed by the local research committee (COV) of the Medical Centre Leeuwarden and a written waiver was obtained as the study was not within the scope of the Medical Research Involving Human Subjects Act (WMO 15-03-2022).

## Results

### Patient characteristics

In total 400 patients were included: 200 pre- and 200 post-intervention. Patient characteristics are shown in Table 2. There was no difference between patient characteristics before and after the implementation of the intervention, with the exception of the distribution across the different departments. Most patients (> 70%) in both groups were admitted to the department of internal medicine, neurology, or pulmonary medicine.

**Table 2:** Patient characteristics.

Patient characteristic	Pre-intervention [n = 200]	Post intervention [n = 200]
Male sex, n [%]	102 [51]	101 [51]
Age in years, median [IQR]	76 [67-83]	74 [66-81]
Weight in kg, median [IQR]	78 [65-90]	77 [65-81]
Body mass index in kg/m^2^, median [IQR]	26 [23 –30]	26 [23 –31]
Department, n [%]		
Internal medicine	63 [32]	64 [32]
Neurology	57 [29]	57 [29]
Pulmonary medicine	23 [12]	48 [24]
Geriatrics	26 [13]	21 [11]
Cardiology	16 [8]	8 [4]
Remaining departments	15 [8]	2 [1]

### Effect of the CDS tool

**Fig 1:**
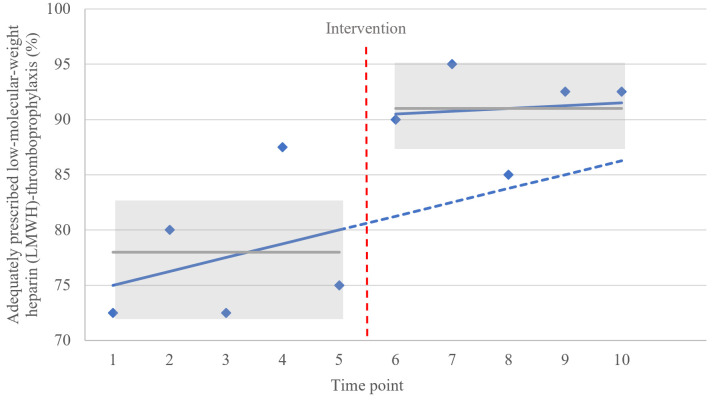
Adequately prescribed thromboprophylaxis pre- and post-intervention. The grey line shows the average percentage pre- and post-intervention with the 95% CI in the grey transparent frame, the solid blue line shows the pre- and post-intervention slopes and the dashed post-intervention line shows the counterfactual trend line. Y-axis starts at 70% to illustrate detailed trends.

Before implementation of the CDS tool, the overall percentage of adequately prescribed thromboprophylaxis was 78%, after implementation of the CDS tool this percentage increased by 13% (95% CI: 6%−20%) to 91%. Although no clear pre-intervention time trend was observed (Fig 1), the intervention effect diminished when accounting for the pre-intervention slope and autocorrelation between time points to 8% (95% CI: −4%−19%) (Table 3). After implementing the CDS tool, besides the direct level effect, the percentage of adequately prescribed thromboprophylaxis did not increase further over time.

**Table 3:** The adjusted effect of the CDS tool on the percentage of adequately prescribed thromboprophylaxis.

	Effect estimate [95% CI]
Slope pre-intervention	1.4% [−1.8%−4.6%]
Slope post intervention	0.4% [−2.7%−3.6%]
Level effect	7.5% [−4.4%−19.5%]

Effect estimates from the interrupted time series analysis using an autoregressive integrated moving average (ARIMA) model, adjusted for the pre-intervention time trend and autocorrelation, are presented.

## Discussion

The implementation of a clinical decision support (CDS) tool integrated into the electronic health record (EHR) was associated with an increase in the percentage of adequately prescribed low-molecular-weight heparin (LMWH) thromboprophylaxis in non-surgical patients at high risk of venous thromboembolism (VTE). While a crude increase of 13% was observed, the effect estimate decreased to 8% after adjustment for pre-intervention trends and autocorrelation.

In this study, before implementation of the CDS tool, an average percentage of 78% adequately prescribed thromboprophylaxis was found, which was higher than expected based on previous literature [6,16,25–28]. In a recent Dutch study, adherence to the thromboprophylaxis guideline of 55% was observed for non-surgical patients with a high VTE risk. An international cross-sectional study performed in 32 countries reported that on average 40% of non-surgical high-risk patients received adequate thromboprophylaxis. The reason for the high percentage in our hospital is not exactly known, but we hypothesize it might be due to increased awareness because of several reported local incidents of VTE in the years prior to performing this study due to inadequate thromboprophylaxis and because of the COVID pandemic when there was a lot of attention for thromboprophylaxis [29]. As a consequence, the potential for further improvement was limited, which likely contributed to the modest effect size observed in this study, consistent with the findings reported by Spyropoulos et al [30].

Several studies have evaluated the impact of CDS tools integrated into EHR systems to improve thromboprophylaxis prescribing. Early work by Kucher et al. demonstrated that electronic alerts to physicians significantly increased thromboprophylaxis use and reduced symptomatic VTE events, although this study was conducted in a setting with substantially lower baseline adherence and did not account for underlying temporal trends [25]. More recent studies have confirmed that alert-based CDS interventions are associated with improved guideline adherence but reported effect sizes vary widely depending on study design, baseline adherence, and whether the intervention was part of a multifaceted quality improvement strategy [6,22–24,28,31]. Systemic reviews and meta-analyses emphasize substantial heterogeneity across studies and highlight that improvements in prescribing behavior do not necessarily translate into measurable reductions in clinical outcomes such as VTE or bleeding events [22–24]. In line with this literature, our study focused on prescribing behavior as a process measure and did not assess clinical outcomes.

With regard to the performance the implemented CDS tool had a sensitivity of 92% and a specificity of 68%. Sensitivity is the ability of a tool to alert prescribers adequately when patients are at risk of an event, where specificity is the ability to correctly identify patients that are not at risk where no alert has to be generated. The ideal CDS tool should have high specificity and sensitivity, however CDS systems tend to have a high sensitivity and lower specificity as we saw with our CDS tool [32–34]. To obtain a high sensitivity an increased number of alerts should be accepted, this can however lead to alert fatigue and overriding of advices generated by the CDS system or patients unnecessarily receiving thromboprophylaxis with higher risk of bleeding as a consequence [35–37]. The ability of a tool to identify as many patients that are at risk against the risk of false positives and overalerting should be carefully weighted. We observed that often prescribers do not immediately follow-up on the advice, which is believed being due to the fact that the pop-up appears when opening the patient file before the prescriber has assessed the patient. We know that appropriate timing of alerts within the workflow is important, alerting as early as possible and at that time having complete information [33–35,37–40].

A limitation of the implemented CDS tool is that bleeding risk was not incorporated and had to be assessed separately by the prescriber. Although no safety signals or reported cases of misprescription attributable to the CDS tool were identified during the study period, bleeding outcomes were not systematically assessed. Balancing thrombotic and bleeding risks remains a key challenge in thromboprophylaxis decision-making, and future versions of the CDS tool may benefit from integrating validated bleeding risk assessment models to improve specificity and safety.

A strength of this study is that a single intervention, a CDS tool, was studied rather than a multifaceted intervention. Because data were collected at different a priori set time points over a longer period, potential underlying trends could be detected and accounted for. However, because an interrupted time series of aggregated level data was used (i.e., percentage of adequately prescribed thromboprophylaxis per month), this analysis has much less statistical power than a straightforward pre-post comparison. Due to the small number of time points some influential periods resulted in a slight positive slope before the intervention, which diminished the direct intervention effect. Moreover, the percentage of adequately prescribed thromboprophylaxis before the intervention was much higher than expected, resulting in less room for improvement. It is known that ITS designs are often underpowered and when the expected effect size is small or the number of time points is small this analysis should be used with caution [41,42].

Further, the optimal performance of the CDS tool is highly dependent on an updated patient file. Risk factors of the PPS have to be documented and kept up at the right locations, for example in the so-called problem list, for the tool to automatically abstract them to be able to give a reliable advice. When risk factors are not documented properly they may be missed by the tool and a patient can incorrectly be seen as a low risk patient. Several studies have found that completeness of the problem list is an issue across healthcare facilities, influencing the performance of CDS tools [43,44]. In our experience, correct definition and registration of reduced mobility, one of the highest scoring risk factors of the PPS (score of 3), is the most challenging and should be given attention.

Future studies should look into the generalizability, safety and effectiveness of this type of intervention across different hospital settings and assessing their impact on clinical outcomes. We aim to further optimize our CDS tool by improving specificity to prevent alert fatigue and over-treatment, and determine the appropriate timing of the alert.

## Conclusion

Our CDS tool integrated in the EHR may increase the percentage of high-risk patients adequately treated with thromboprophylaxis. With increasing amounts of data available in electronic health records, fine-tuned CDS tools might be an efficient and sustainable intervention to improve healthcare quality without interrupting workflow. Future research is needed to further optimize these types of tools to reach their full potential.

## Supporting information

S1 Dataset Supporting dataset. (XLSX)
